# Regulation of lipid metabolism by the unfolded protein response

**DOI:** 10.1111/jcmm.16255

**Published:** 2021-01-04

**Authors:** Matthieu Moncan, Katarzyna Mnich, Arnaud Blomme, Aitor Almanza, Afshin Samali, Adrienne M. Gorman

**Affiliations:** ^1^ Apoptosis Research Centre School of Natural Sciences National University of Ireland Galway Ireland; ^2^ Laboratory of Cancer Signaling GIGA‐institute University of Liège Liège Belgium

**Keywords:** activating transcription factor 6, cholesterol, endoplasmic reticulum, fatty acid, inositol‐requiring enzyme 1, lipid metabolism, phospholipid, PRKR‐like endoplasmic reticulum kinase, triglyceride, unfolded protein response

## Abstract

The endoplasmic reticulum (ER) is the site of protein folding and secretion, Ca^2+^ storage and lipid synthesis in eukaryotic cells. Disruption to protein folding or Ca^2+^ homeostasis in the ER leads to the accumulation of unfolded proteins, a condition known as ER stress. This leads to activation of the unfolded protein response (UPR) pathway in order to restore protein homeostasis. Three ER membrane proteins, namely inositol‐requiring enzyme 1 (IRE1), protein kinase RNA‐like ER kinase (PERK) and activating transcription factor 6 (ATF6), sense the accumulation of unfolded/misfolded proteins and are activated, initiating an integrated transcriptional programme. Recent literature demonstrates that activation of these sensors can alter lipid enzymes, thus implicating the UPR in the regulation of lipid metabolism. Given the presence of ER stress and UPR activation in several diseases including cancer and neurodegenerative diseases, as well as the growing recognition of altered lipid metabolism in disease, it is timely to consider the role of the UPR in the regulation of lipid metabolism. This review provides an overview of the current knowledge on the impact of the three arms of the UPR on the synthesis, function and regulation of fatty acids, triglycerides, phospholipids and cholesterol.

## 1 INTRODUCTION

The endoplasmic reticulum (ER) is one of the largest organelles in the cell. It is involved in multiple fundamental biological processes including protein folding and secretion, Ca^2+^ storage and lipid synthesis. In response to stress (eg accumulation of misfolded proteins), the ER triggers the unfolded protein response (UPR), a complex and conserved signalling pathway that is mediated by three ER transmembrane sensor proteins: inositol‐requiring enzyme 1 alpha (IRE1α), protein kinase RNA‐like ER kinase (PERK) and activating transcription factor 6 (ATF6). Once active, these sensors induce an elaborate and integrated signalling network that either allows restoration of ER homeostasis or triggers cell death.^1^

In addition to protein folding, the ER plays an important role in the regulation of lipid metabolism. Several enzymes involved in triglyceride (TG) and cholesterol biosynthesis, as well as enzymes participating in the regulation of membrane turnover and dynamics, are located in the ER. Consequently, activation of the UPR has important implications for lipid and sterol synthesis, some of which are yet to be unravelled. The purpose of this review is to describe the role of the UPR in the regulation of lipid metabolism, in order to introduce this complex field to a new audience. We have focussed on primary pathways including the synthesis, function and regulation of fatty acids (FAs), TGs, phospholipids (PLs) and cholesterol, and describe their regulation by the UPR.

## 2 THE UNFOLDED PROTEIN RESPONSE

### 2.1 Role and activation

IRE1α, PERK and ATF6 share a common architecture, which consists of an ER luminal N‐terminal sensing domain, a transmembrane region and a cytosolic domain that is involved in signal transduction. Normally inactive, IRE1α, PERK and ATF6 are activated by various cellular stresses, including glucose deprivation, disruption of calcium homeostasis, excessive production of reactive oxygen species, viral infection, hypoxia and altered proteasome activity, which result in ER stress through the accumulation of misfolded proteins.^1^ Under basal conditions, the luminal domains of IRE1α, PERK and ATF6 are bound to binding‐immunoglobulin protein (BiP, also known as GRP78), a chaperone protein involved in the folding of nascent polypeptides within the ER. When misfolded proteins accumulate in the ER lumen, BiP dissociates from the three ER stress sensors, enabling their activation. IRE1α and PERK can also be activated directly by binding of misfolded proteins to their ER luminal domains.^2^, ^3^ UPR activation leads to the transient expression of specific sets of genes involved in the folding, synthesis and degradation of proteins.

However, these sensors can also be activated in response to ER membrane perturbations caused by changes in PL composition,^4^ in cholesterol,^5^ sterol^6^ and inositol^7^ levels and by changes in lipid accumulation^8^ and saturation.^9^ For example, in the absence of misfolded proteins, IRE1α and PERK can still respond to the lipid bilayer stress induced by increased lipid membrane saturation,^9^ while ATF6 can be activated by specific species of sphingolipids.^4^ More details on the mechanisms governing the activation of the UPR sensors are reviewed elsewhere.^1^

### 2.2 *IRE1*α

**FIGURE 1:**
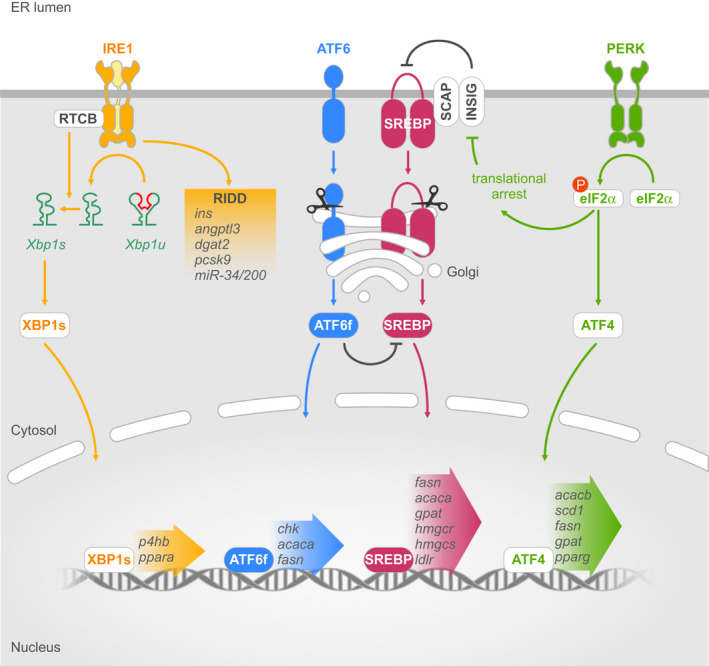
The Unfolded Protein Response (UPR) is controlled by three endoplasmic reticulum (ER) stress sensors: inositol‐requiring enzyme 1 (IRE1), activating transcription factor 6 (ATF6) and PKR‐like ER kinase (PERK). Upon activation, IRE1 splices x‐box binding protein 1 (XBP1) mRNA, which is then ligated by RTCB and translated into XBP1s. IRE1 also cleaves cytosolic RNA in the process called regulated IRE1 dependent decay (RIDD), which reduces levels of target transcripts. Activated ATF6 is translocated to the Golgi, where its N‐terminal fragment is released by proteases and translocated to the nucleus. PERK‐mediated phosphorylation of eIF2α inhibits a global expression of genes, like SREBP activity‐regulating Insig, and prompts selective translation of ATF4. The figure shows genes involved in lipid metabolism that are regulated by each of the three transcription factors, XBP1s, ATF6f and ATF4, produced by the UPR

IRE1 is the most evolutionarily conserved UPR sensor. IRE1α is expressed ubiquitously while its paralog, IRE1β, is restricted to the lungs and gastrointestinal tract.^1^ IRE1α possesses two distinct enzymatic activities that are mediated by cytosolic kinase and RNase domains.^10^ Upon activation during ER stress, IRE1α forms homodimers and oligomers. This enables the *trans*‐autophosphorylation of IRE1α's kinase domain leading to the allosteric activation of its RNase domain.^11^ IRE1α's RNase domain catalyses the excision of 26 nucleotides from X‐box binding protein 1 (*XBP1*) mRNA and produces a frameshift that allows the translation of a longer isoform called spliced XBP1 (XBP1s) (Figure 1).^12^ XBP1s is a transcription factor that induces the expression of genes involved in lipid synthesis, chaperone protein synthesis and ER‐associated degradation (ERAD) machinery, leading to increased ER size and capacity.^1^ In contrast, unspliced XBP1 (XBP1u) lacks transcriptional activity. IRE1α's RNase activity also facilitates the degradation of various mRNAs, cleaving them at a defined consensus sequence through a process called regulated IRE1‐dependent decay (RIDD) (Figure 1).^1^ Identification of IRE1α mRNA targets revealed that RIDD activity can reduce the load of newly synthesized peptides entering into the ER, or promote apoptosis.^1^, ^13^ The kinase activity of IRE1α is associated with the activation of both the JNK and the NF‐κB pathways, resulting in increased autophagy and apoptosis.^14^, ^15^

### 2.3 PERK

Similar to IRE1α, PERK also oligomerises and *trans*‐autophosphorylates following activation by ER stress. Once activated, the cytosolic kinase domain of PERK phosphorylates and inactivates eIF2α, an essential component of the 43S pre‐initiation complex necessary for the initiation of cap‐dependent protein translation (Figure 1). This leads to a global arrest in protein translation.^1^ At the same time, eIF2α phosphorylation allows translation of a specific set of mRNAs that carry one or more upstream open reading frames in their 5′ untranslated regions.^16^ Thus, activation of the PERK pathway has a dual function: decreasing the entry of newly synthesized peptides into the ER to alleviate ER stress while simultaneously stimulating the production of proteins that are critical for stress adaptation.^1^, ^16^ The latter process is exemplified by the specific translation of ATF4, an important transcription factor that plays key roles in autophagy, antioxidant response, amino acid metabolism and the synthesis of stress‐induced proteins.^16^

### 2.4 ATF6

Unlike IRE1α and PERK, ATF6 activation does not involve phosphorylation. Release of ATF6 from BiP exposes Golgi‐localization sequences present on the luminal domain of ATF6.^17^ Once transported into the Golgi, site‐1 (S1P) and site‐2 proteases (S2P) cleave ATF6 and release a cytosolic fragment containing a basic leucine zipper (bZIP) transcription factor called ATF6f (Figure 1).^18^, ^19^ ATF6f induces expression of *XBP1* as well as genes involved in protein folding, ERAD machinery, ER homeostasis and ER and Golgi biogenesis.^20^, ^21^ ATF6f and XBP1s can form heterodimers, whose association induces expression of ERAD proteins.^22^

## 3 REGULATION OF LIPID METABOLISM BY THE UPR

### 3.1 Overview of lipid metabolism

**FIGURE 2:**
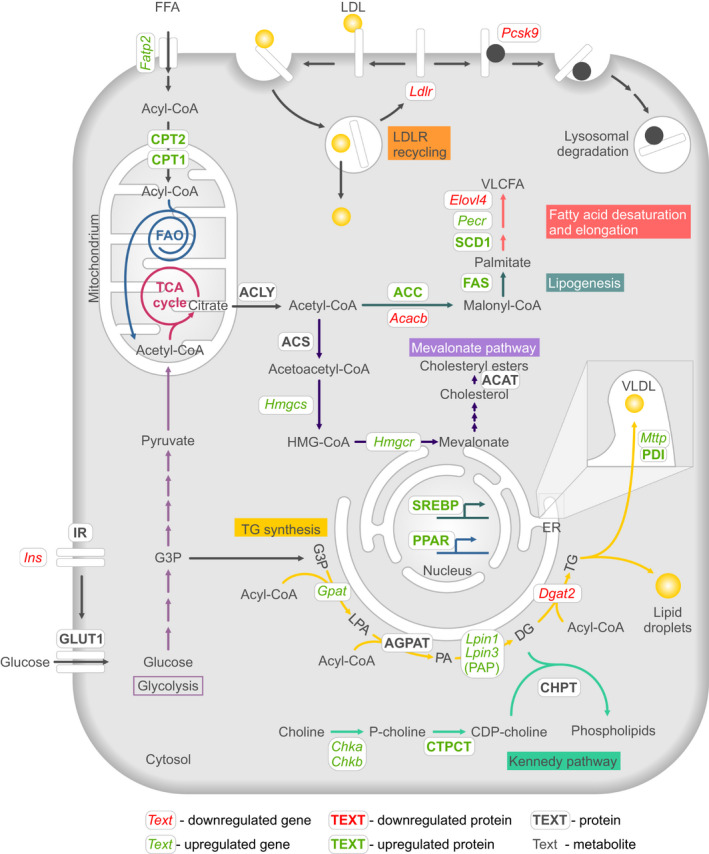
Overview of the major lipid metabolic pathways regulated by the UPR: Kennedy pathway, TG synthesis, lipogenesis, mevalonate pathway, fatty acid elongation and desaturation, fatty acid oxidation, tricarboxylic acid cycle, very low‐density lipoprotein formation and low‐density lipoprotein uptake. Only key nodes are shown. For further details, see the main text. Abbreviations: ACC, Acetyl‐CoA carboxylase; ACACB, Acetyl‐CoA carboxylase 2; ACAT, acyl‐CoA:cholesterol acyltransferase; ACLY, ATP‐citrate lyase; ACS, Acetyl‐CoA synthetase; CoA, coenzyme A; AGPAT, Acylglycerol‐P acyltransferase; CHKA, Choline kinase alpha; CHKB, Choline kinase beta; CHPT, Cholinephosphotransferase; CPT, Carnitine palmitoyltransferase; CTPCT, Phosphocholine cytidylyltransferase; DG, Diacylglycerol; DGAT2, DG acyltransferase; ELOVL4, Elongation of very long chain fatty acids‐4; ER, Endoplasmic reticulum; FATP2, Fatty acid transport protein 2; FFA, Free fatty acid; FAO, Fatty acid oxidation; FAS, Fatty acid synthase; G3P, Glycerol‐3 phosphate; GLUT1, Glucose transporter 1; GPAT, Glycerol‐P acyltransferase; HMGCR, HMG‐CoA reductase; HMGCS, HMG‐CoA synthase; Ins, insulin; IR, insulin receptor; LDL, Low‐density lipoprotein; LDLR, LDL receptor; LPIN, Lipin; LPA, Lysophosphatidic acid; MTTP, Microsomal TG‐transfer protein complex; PA, phosphatidic acid; PAP, Phosphatidic acid phosphohydrolase; PCSK9, Proprotein convertase subtilisin/kexin type 9; PECR, Peroxisomal trans‐2‐enoyl‐CoA reductase; PDI, Protein disulphide isomerase; PPAR, Peroxisome proliferator‐activated receptor; SCD1, Stearoyl‐CoA desaturase‐1; SREBP, Sterol regulatory element‐binding protein; TCA, tricarboxylic acid cycle; TG, Triglycerides; VLCFA, Very long chain fatty acids; VLDL, Very low‐density lipoproteins

Lipid molecule in cells can be subdivided into two large groups: the long hydrocarbon chain‐containing FAs, including TGs and PLs, and the ring‐structured sterols. FAs are composed of a carboxyl group linked to a long aliphatic hydrocarbon chain that is either saturated or unsaturated. De novo FA synthesis occurs through a cellular process called lipogenesis (Figure 2) and FAs provide the building blocks for the formation of TGs and PLs. Energy stored in TGs can be released in a catabolic process called β‐oxidation. TGs usually coalesce into lipid droplets before being secreted into the blood as very low‐density lipoproteins (VLDL), usually by hepatocytes (Figure 2). VLDLs can then either enter adipocytes, where TGs will be stored, or be transported to other cell types, where they support energy production.^23^ In contrast, PLs are the main components of cell membranes and can fulfil structural (eg phosphatidylethanolamine [PtdEtn], phosphatidylcholine [PtdCho], sphingomyelin [SM] and phosphatidylserine [PtdSer])^24^ and signalling functions (eg phosphatidylinositol [PtdIns]).^25^ Among them, PtdEtn and PtdCho are the most abundant lipids in cell membranes.

The main sterol found in animals is cholesterol. Cholesterol consists of a four‐ringed sterol structure that can either be absorbed from the diet or synthesized endogenously. Within cell membranes, cholesterol levels influence membrane fluidity. Cholesterol can also act intracellularly as a precursor for steroid hormones, oxysterols and bile acids.^26^ To avoid high levels of circulating free cholesterol in the blood, the latter is esterified to cholesteryl esters by acyl‐CoA:cholesterol acyltransferase (ACAT) (Figure 2), which can be stored in lipid droplets or secreted into lipoproteins such as chylomicrons, in a manner that is similar to TGs.^27^

FAs, TGs, PLs and cholesterol all originate from acetyl‐CoA, a glucose‐derived metabolite that plays a central role in oxidative phosphorylation (Figure 2). Acetyl‐CoA is made available for lipid and cholesterol synthesis via the cleavage of a citrate molecule, which is transported across the mitochondrial membrane to the cytoplasm where it is cleaved into oxaloacetate and acetyl‐CoA by the ATP‐citrate lyase (ACLY) (Figure 2).^28^ Once in the cytoplasm, acetyl‐CoA is used for the synthesis of more complex lipid molecules. Two carbons are sequentially added in a repeated manner to an acetyl‐CoA backbone in a series of reactions catalysed by the acetyl‐CoA‐carboxylase (ACC)^29^ and fatty acid synthase (FASN) (Figure 2). The product of this reaction is palmitic acid, a 16‐carbon saturated FA, which can be elongated to produce very long chain FAs (VLCFA).^30^

Addition of FAs to coenzyme A produces FA‐CoA molecules, which are used to generate both glycero‐ and phospholipids (Figure 2). Glycerol‐P acyltransferase (GPAT) catalyses the attachment of the first FA‐CoA to a glycerol‐3 phosphate (G3P) backbone, producing a monoacylglycerol molecule also called lysophosphatidic acid (LPA). Acylglycerol‐P acyltransferase (AGPAT) adds a second FA‐CoA to LPA, converting it into phosphatidic acid (PA). PA phosphohydrolase (PAP) removes the phosphate group on the third alcohol of the PA molecule to produce diacylglycerol (DG).

DG is at the branch‐point between TGs and PLs, and the generation of TGs or PLs from FAs is context dependent (Figure 2).^31^ TGs are produced by the addition of a third FA‐CoA to DG by the enzyme DG acyltransferase (DGAT). When PLs synthesis is favoured, a member of the DG kinase (DGK) family can reverse the action of PAP, converting DG back to PA by adding a new phosphate group to DG.^32^ This process enables the newly synthesized PA to enter into the cellular pool of PLs, where it can undergo further modification and contribute to the synthesis of new membranes or to replenish the levels of signalling lipids. A well‐described example of PL synthesis from PA is the Kennedy Pathway,^33^ which describes the production of PtdEtn and PtdCho from ethanolamine and choline, respectively (Figure 2).

Despite also relying on acetyl‐CoA, cholesterol synthesis is synthesized through a different multistep metabolic pathway termed mevalonate pathway, involving more than 15 enzymes and 30 different reactions.^34^ Here, we have focussed on the limiting steps of that pathway such as the rate of cholesterol synthesis, which is mediated by the activities of the HMG‐CoA synthase (HMG‐CS) and the HMG‐CoA reductase (HMG‐CR), two enzymes whose expressions are tightly regulated by lipid metabolism (Figure 2).^34^

### 3.2 Classical known regulators of lipid metabolism

Accumulation of lipid intermediates in non‐adipose cells often has detrimental effects for cell function, a phenomenon known as lipotoxicity. To prevent such toxicity, eukaryotic cells have developed control mechanisms to regulate lipid metabolism. A major component of this regulation system is the SREBP‐SCAP‐Insig pathway. Similar to ATF6, sterol regulatory element‐binding proteins (SREBPs) are ER‐resident proteins possessing transcription factor activity. Release of the functional transcription factor requires the processing of the native SREBP1 form in the Golgi apparatus by S1P and S2P^35^ to activate the transcription of a large number of genes involved in the synthesis of FA derivatives and cholesterol (Figure 1).^36^ These include, among others, *FASN*,^37^ *ACACA*,^38^ *LDLR*,^39^ *HMGCS*,^40^ *HMGCR* ^41^ and *GPAT*.^42^ The SREBP family is composed of three proteins SREBP1a, SREBP1c and SREBP2, encoded by two different genes: *SREBP1* and *SREBP2*. SREBP1a is highly expressed in intestinal epithelial cells, cardiomyocytes, macrophages and bone marrow dendritic cells, and has a high potency for stimulating both lipogenic and cholesterogenic gene expression.^43^ In contrast, SREBP1c is predominant in most tissues and acts mainly by controlling the expression of lipogenic genes.^44^ Thus, appropriate SREBP1c activity is critical for the regulation of FAs and TGs in lipogenic cells such as hepatocytes and adipocytes.^45^ SREBP2 expression has been confirmed in a large variety of tissues. SREBP2 mainly mediates sterol regulation and is therefore complementary to SREBP1c.^36^ Regulation of SREBP activation is dependent on SREBP cleavage‐activating protein (SCAP) and Insulin Induced Gene (Insig). SCAP is an escort protein that allows SREBPs to enter into the Golgi‐targeted COPII coated vesicles through to its MELADL motif.^46^ Insig, which is directly bound to SCAP, maintains the SREBP‐SCAP protein complex in the ER membrane (Figure 1). SCAP has the ability to detect the presence of cholesterol,^47^ while Insig, which can be induced by insulin in the liver,^48^ is able to sense oxysterols. Hence, the presence of cholesterol and oxysterol promotes the binding of SCAP and Insig, ultimately inhibiting the SREBP pathway.^46^, ^49^, ^50^

Carbohydrate‐responsive element‐binding protein (ChREBP), peroxisome proliferator‐activated receptor (PPAR) γ and CCAAT/enhancer‐binding protein (C/EBP) α are also transcriptional regulators of lipid and sterol synthesis. ChREBP is a glucose‐responsive transcription factor^51^ and can induce the transcription of genes such as the liver pyruvate kinase (*PKL*), which converts phosphoenolpyruvate (PEP) to pyruvate during glycolysis, the glucose transporter *GLUT4*, the glycerol‐3‐phosphate dehydrogenase (*GPD*), which participates in the production of G3P, and the lipogenic genes *ACLY*, *FASN* and *ACAC*.^52^, ^53^ Hence, ChREBP is an important transcription factor in both glucose and lipid metabolism. PPARs are nuclear receptors that are able to induce gene transcription in response to FA binding. Three distinct isoforms of PPAR exist: α, δ and γ. Among them, PPARγ has the most important role in lipogenesis. It is highly expressed in adipocytes and has a predominant role in adipocyte differentiation and lipid storage.^54^, ^55^ PPARα is expressed in various tissues like the liver, the heart and muscles, and is described to control FA catabolism.^56^, ^57^ Finally, PPARδ is ubiquitously expressed and is involved in various functions such as wound healing and VLDL signalling in macrophages.^58^, ^59^ C/EBPα is a transcription factor, which is also highly expressed in liver and adipose tissues.^60^, ^61^ It has been linked to the expression of well‐described lipid metabolism regulators, namely *PPARA*, *SREBP1* and *ChREBP*, as well as two gluconeogenic enzymes (phosphoenolpyruvate carboxykinase and glucose‐6‐phosphatase).^62^, ^63^, ^64^

### 3.3 Regulation of lipid metabolism by IRE1α

Both the XBP1s and RIDD pathways modulate the expression of genes involved in lipid synthesis, thus supporting the critical role of IRE1α in the regulation of lipid metabolism. A transcriptional study highlighted a set of lipid‐related genes activated in response to overexpression of human XBP1s in mouse fibroblasts.^65^ Lipin genes (*Lpin1* and *Lpin3*) were among the most up‐regulated metabolic genes together with the *Osbp* gene, which encodes a sterol‐sensing protein that modulates SREBP activity in response to sterol,^66^ *Pecr*, an enzyme involved in FA elongation,^67^ *Lss*, which catalyses the formation of lanosterol from squalene^68^ and *Gpat4*, an enzyme which adds a FA to glycerol during lipogenesis (Figure 2).^69^ The gene encoding FA elongase 4, *Elovl4*, was down‐regulated. Another study demonstrated that conditional postnatal KO of *Xbp1* in mice liver led to impairment of both FA and sterol synthesis in hepatocytes.^70^ Impaired lipogenesis was associated with down‐regulation of *Scd1*, *Dgat2* and *Acacb*, with no significant changes in SREBP targets. Specific binding of XBP1s to the regulatory sequences of these genes was confirmed by ChIP in liver nuclear extracts from mice injected with tunicamycin or fed a high‐fructose diet. However, XBP1s overexpression in primary mouse hepatocytes was insufficient to re‐activate *Scd1* expression, in contrast to *Dgat2* and *Acacb*.^70^

The IRE1α/XBP1s axis has also been studied in the context of ER expansion in plasmocytes,^71^ pancreatic acinar cells and salivary gland cells.^72^ A series of reports by Brewer and colleagues investigated the interactions between the UPR and the Kennedy pathway in NIH‐3T3 fibroblasts, and how these two pathways interact to activate ER membrane biogenesis.^65^, ^73^ Their data highlighted a role for XBP1s in the promotion of PtdCho and PtdEtn synthesis, which is needed for ER expansion. The authors demonstrated that the primary mechanism by which overexpression of human XBP1s led to a higher production of PtdCho was through an enhanced activity of phosphocholine cytidylyltransferase (CTPCT) in Kennedy pathway. They further proposed that this increase in activity occurred through protein up‐regulation and was independent of gene transcription, suggesting the existence of post‐transcriptional or post‐translational mechanisms of regulation. However, increased expression of CTPCT could also be the result of the global increase in protein synthesis that was observed following hXBP1s overexpression.^71^ In addition, hXBP1s overexpression did not affect the levels of cholesterol, cholesteryl esters or TGs, which contrasted with the reported increase in FA synthesis.

These observations echo a recent publication describing the murine hepatic response to refeeding. In liver‐specific *Xbp1* KO (*Xbp1* LKO) mice, hepatic XBP1s expression was required for the translation, but not transcription, of de novo lipogenesis genes such as *Srebp1c*, *Fasn* and *Acac*.^74^ This effect was linked to a phospho‐eIF2α‐induced arrest in global translation, leading to the conclusion that, while XBP1s might not be necessary for the transcriptional induction of lipogenic genes, its activity is necessary to manage fasting and refeeding states in the liver.^74^ The same publication showed that *Xbp1s* LKO mice had decreased plasma levels of TGs, due to an impaired secretion by the liver, in both fasting and refeeding states. This strengthened the idea that the function of XBP1s in hepatic lipid metabolism is not restricted to lipogenesis.^74^ A similar observation was made during the characterization of a conditional postnatal *Xbp1* KO in mice liver, in which decreased levels of plasma TGs, cholesterol and free FAs were observed in KO animals compared to controls.^70^ A possible explanation for these results came from a later study which showed that XBP1 overexpression was able to revert deficient secretion of TGs in hepatocytes from liver‐specific *Ern1a* KO mice by increasing protein disulphide isomerase (PDI) expression.^75^ PDI is a subunit of the microsomal TG‐transfer protein complex (MTP) which is essential for normal MTP activity,^76^ the latter being required for proper VLDL biogenesis.^77^ Additionally, activation of the IRE1α‐XBP1 pathway was reported in mice during prolonged fasting or under ketogenic diet.^78^ Liver‐specific deletion of *Ern1a* led to the impairment of β‐oxidation and ketogenesis in a process that is dependent on the XBP1‐induced up‐regulation of PPARα, thus indicating a role for IRE1α and XBP1 in the regulation of metabolic adaptive programs.

Involvement of XBP1 in lipid metabolism has also been suggested to be tissue specific. Indeed, XBP1 expression in murine adipocytes was induced during lactation in vivo and in response to prolactin treatment in vitro, demonstrating that XBP1s activation could be hormone‐responsive.^79^ Adipocyte‐specific deletion of XBP1 in mice further resulted in increased body weight during lactation and reduced milk production, despite no change in the milk lipid composition. Overexpression of XBP1s in F442A adipocytes led to a down‐regulation of several lipogenic genes, such as *Scd1*, *Fas*, *Acc*, *Dgat1*, *Dgat2* and *Lpl*.^79^ Prolactin treatment of *Xbp1*‐deficient adipocytes increased expression of the SCD1 protein. Similar results were observed in adipose tissue from XBP1‐deleted dams during lactation, which displayed higher levels of both FAS and SCD1 proteins. Consequently, the authors proposed that XBP1 expression in adipocytes could induce a state of low lipogenic activity in response to prolactin, ultimately promoting a shift in lipid usage from adipocytes to mammary gland during lactation.

A number of observations suggest a role for RIDD in the regulation of lipid metabolism. These include IRE1α‐dependent degradation of *Ins* mRNA in rat insulinomia cells and IRE1β‐dependent degradation of *Mttp* mRNA in mouse intestinal and Huh7 cells.^80^, ^81^ However, inducible genetic deletion of XBP1 has been linked to hyperactivation of IRE1α, thus making it difficult to uncouple the roles of XBP1 and RIDD.^70^ Thus, some observations made upon genetic manipulation of XBP1 could be due to increased RIDD activity. The contribution of XBP1 and RIDD activity to hepatic lipid metabolism was examined in mice carrying liver‐specific deletions of *Ern1a* or *Xbp1*. Impairment of RIDD activity, through IRE1α silencing, partially restored TG and cholesterol levels in the liver of *Xbp1* LKO mice. Partial reversion of the phenotype was accompanied by an increase in *Dgat2* and *Acacb* mRNA expression, suggesting these genes are potential RIDD targets.^82^ In addition, the mRNA for proprotein convertase subtilisin/kexin type 9, an enzyme involved in the clearance of the LDL receptor, and for Angptl3, an inhibitor of TG hydrolysis, were also proposed as potential RIDD targets.^82^ RIDD targets also include miRNAs. For example,*miR‐34* and *miR‐200*, two miRNAs that are known to down‐regulate PPARα and SIRT1 expression in mouse liver,^83^ have also been identified as RIDD targets.^56^, ^84^ Furthermore, a recent paper demonstrating unconventional IRE1‐dependent maturation of miR‐2137 in macrophages links RIDD with phosphatidylinositide‐derived signalling lipid metabolites and downstream signalling by mTOR, since phosphatidylinositol (3,4,5) phosphate (PI(3,4,5)P3) 5‐ phosphatase‐2 (INPPL1) is a direct target of miR‐2137.^85^

Consequently, IRE1α seems to regulate lipid metabolism through both XBP1 and RIDD. XBP1 seems to play an essential role in the transport of TGs from the liver to adipocytes, and in the induction of PPARα‐mediated lipolysis. By contrast, IRE1α's RIDD activity seems to promote lipid hydrolysis and prevent lipid storage, both by reducing the expression of lipogenic genes and lipolysis inhibitors. Nevertheless, their roles are not fixed as demonstrated by XBP1s overexpression in *C* *elegans* which reduced TG and increased lipase activity.^86^

### 3.4 Regulation of lipid metabolism by PERK

PERK signalling affects lipid homeostasis through its downstream targets eIF2α, ATF4 and CHOP. Insig‐1 has been reported to be particularly sensitive to PERK‐induced translational pausing, and its PERK‐dependent depletion is a major step in the activation of lipogenesis in response to PERK activation. In fact, the absence of PERK in murine mammary epithelium caused a reduction in TG and FA content in milk. Insig‐1 depletion in WT MEFs induced the processing of SREBP in a PERK‐ and eIF2α‐dependent manner. In contrast, this process, as well as the induction of SREBP, FASN, SCD1 and ACL expression, was blocked in *Perk* KO MEFs and mammary glands.^87^ A similar observation was made in CHO‐7 cells subjected to hypotonic stress.^88^ Despite treatment of cells with various cholesterol derivatives or LDL to inhibit SREBP proteolytic activation, hypotonic stress‐induced SREBP cleavage due to Insig‐1 depletion. In support of these findings, the ER stressor thapsigargin induced both the depletion of Insig‐1 and the stimulation of SREBP processing. However, while the transcriptional activity of SREBP through increased expression of *Hmgcs* and *Hmgcr* was confirmed, increased FA or cholesterol synthesis was not observed (see below). Therefore, it was hypothesized that this effect could be due to PERK‐induced translational arrest of SREBP‐regulated genes. Finally, in another study using cytomegalovirus (HCMV)‐infected primary human fibroblasts, it was reported that Insig‐1 levels and SREBP1 activation modulated by PERK were independent of eIF2α phosphorylation, suggesting the involvement of other PERK‐dependent mechanisms in SREBP1 activation.^89^ Additionally, overexpression of BiP in ob/ob mice reduced both ER stress and SREBP‐1c cleavage in the liver, reinforcing the idea of the involvement of the UPR in the activation of the SREBP pathway.^90^

A further role for eIF2α in the regulation of gluconeogenesis and lipid synthesis was demonstrated using a mouse model with liver‐specific overexpression of C‐terminal GADD34 fragment, leading to eIF2α dephosphorylation in the liver.^91^ Phenotypically, these mice were more susceptible to fasting hypoglycaemia, as evidenced by an accelerated decrease in blood glucose levels, diminished hepatic glycogen reserves and attenuated gluconeogenesis. Moreover, these mice were less sensitive to high‐fat diet‐induced hepatic steatosis. The authors linked these observations to a decreased expression of C/EBPα and β. Although the precise pathway linking eIF2α‐induced translational pausing to C/EBP isoforms expression was not addressed, they showed that the expression of these transcription factors was responsive to the phosphorylation state of eIF2α. Defective induction of C/EBPα and β resulted in reduced expression of *Pparg* and the PPARγ transcriptional targets *Fasn*, *Acaca*, *Acacb* and *Scd1*. Altogether, defects in C/EBPα and PPARγ could explain both the defects in gluconeogenesis and the low levels of TGs observed in the liver. Moreover, use of a mouse strain carrying an inducible chimeric Fv2E‐PERK kinase domain revealed that activation of PERK‐induced lipo‐ and glycogenic pathways was transient, as the induction of PPARγ and other C/EBP targets responded biphasically to a high activation of the Fv2E‐PERK.^91^ This mechanism could explain the lack of changes in lipid synthesis observed in the CHO‐7 cells subjected to hypotonic stress.^88^

Phosphorylated eIF2α facilitates increased translation of the transcription factor ATF4 whose expression exerts a strong impact on TG homeostasis in mice. With a high‐carbohydrate diet, *Atf4* KO mice displayed reduced hepatic and serum levels of TGs compared to WT mice, as well as glucose and insulin tolerance. While the hepatic transcriptional expression of lipogenic enzymes (*Acac*, *Scd1*, *Fas* and *Gpat*) was induced under high‐carbohydrate diet in both strains, their expression level was significantly lower in *Atf4* KO mice. In contrast, the expression of carnitine palmitoyltransferase 1 (*Cpt1*), a rate‐limiting enzyme in FA β‐oxidation did not vary between the two strains, with both normal and high‐carbohydrate diets. The protective effect of ATF4 deletion against diet‐induced liver steatosis was proposed to be partially linked to *Scd1* loss. Indeed, liver‐specific overexpression of ATF4 in mice increased SCD1 protein expression, while oral supplementation with oleate, the main product of SCD1 activity, increased hepatic lipid accumulation and liver weight in *Atf4* KO mice.^92^ Similar observations were reported in *Atf4* KO mice fed a high‐fructose diet,^93^ where expression of lipogenic enzymes (PPARγ, FAS and ACC) were reduced in the liver, while the expression of proteins involved in FA oxidation (ACOX1, CPT‐1) was unaffected. Hence, the authors concluded that ATF4 deficiency decreased hepatic lipogenesis but did not affect TG secretion and FA oxidation, thus protecting *Atf4*‐deficient mice from fructose‐induced hepatic hypertriglyceridemia.^93^

### 3.5 Regulation of lipid metabolism by ATF6

The contribution of ATF6 to the regulation of lipid metabolism is evidenced by its ability to induce ER expansion in an XBP1‐independent manner.^94^ In NIH‐3T3 cells overexpressing nuclear active form of ATF6 (ATF6f) increased expression of choline kinase isoforms, *Chka* and *Chkb* was reported, which correlated with increased choline kinase and CHPT1 activity and enhanced synthesis of PtdCho.^94^ However, similar to XBP1, this effect was not linked to a transcriptional regulation and was probably due to post‐transcriptional or post‐translational effects. Hence, ATF6 and XBP1 seem to exert complementary effects on the production of PtdCho. Moreover, while a direct regulation has not been confirmed, overexpression of ATF6 also increased the expression of *Acacb* and *Fasn* in MEFs, and increased FA biosynthesis in CHO cells.^94^

In response to glucose deprivation, ATF6 inhibits cholesterol synthesis, exerting its effect through interaction with the processed form of SREBP2, and thus promoting recruitment of the transcription inhibitor HDAC1. This process in turn inhibits SREBP2‐induced lipogenesis in HepG2 cells and leads to the down‐regulation of *HMGCR*, *HMGCS*, *FDFT1* (squalene synthase) and LDLR expression.^95^

In response to direct activation by dihydrosphingosine and dihydroceramide, two intermediates of the sphingolipid/ceramide biosynthetic pathway, ATF6 activates a lipid‐specific transcriptional programme, while thapsigargin treatment induced both lipid and ER stress‐related responses in HEK293 cells.^4^ Further experiments suggest that the role of ATF6 in lipid metabolism is strongly linked to PPARα and its ability to promote FA oxidation. ATF6 deficiency or overexpression of dominant‐negative ATF6 (dnATF6) in mice promoted liver steatosis in response to tunicamycin treatment or high‐fat, high‐sucrose diet, respectively.^96^, ^97^ In contrast, activation of ATF6 by tunicamycin injection in WT mice prevented the accumulation of TGs in the liver through the activation of genes involved in FA oxidation (*Cpt1*, *Cpt2*, *Acox1* and *Ppara*) and in VLDL formation (*Mttp*, *Pdi* and *Apob*).^96^ Mechanistically, ATF6 physically interacts with PPARα/RXRα (retinoid X receptor alpha) heterodimers and is required for their transcriptional activity in mouse liver.^97^ In line with these data, liver‐specific overexpression of dnATF6 blocked PPARα/RXRα transcriptional activity in a luciferase reporter assay. Expression of ATF6 in mice liver also correlated with PPARα expression during fasting, a scenario that enhances TG levels and promotes increased lipid deposition in the liver. Hence, liver‐specific overexpression of dnATF6 in fasted mice caused hepatic steatosis and reduced levels of serum β‐hydroxybutyrate (a marker of β‐oxidation). By contrast, liver‐specific overexpression of ATF6f improved hepatic condition in steatosis‐induced mice fed a high‐fat high‐sucrose diet. In this study, ATF6 did not affect FA synthesis, suggesting that in the liver ATF6 activity might be more important for FA oxidation than synthesis.^97^

ATF6 function has also been assessed in kidney. Using *Atf6*‐deficient mice, a recent study compared the contribution of ATF6 to the development of both liver and kidney steatosis. ER stress induction, following injection of tunicamycin, led to the inhibition of β‐oxidation in the liver of both WT and KO mice strains, although the effects were much stronger in the KO condition. In contrast, renal lipogenesis and β‐oxidation were unaffected, despite a clear lipid accumulation in the kidneys of KO mice. Additionally, WT mice recovered faster than KO mice from tunicamycin treatment, suggesting that ATF6 expression protects against tunicamycin‐induced inhibition of β‐oxidation in the liver, but not the kidneys, of these mice.^98^ Another study, performed in vitro in a human kidney cell line (HK‐2) overexpressing either dnATF6 or ATF6f, also indicated that ATF6 function in kidney is opposite to that observed in liver.^99^ Indeed, ATF6f increased the expression of genes involved in FA uptake (*FATP2*), but decreased the expression of those involved in β‐oxidation (*PPARA* and *CPT2*). These observations were further supported by a murine model of tubulointersititial fibrosis (TIF) in *Atf6*‐deficient mice which showed that TIF formation following unilateral ischaemia reperfusion injury occurred through the activation of ATF6.^99^ In WT mice, ATF6 activation down‐regulated PPARα, causing in turn a reduction in β‐oxidation and an accumulation of lipid droplets, ultimately leading to cell death. By contrast, *Atf6*‐deficient mice displayed higher levels of PPARα, attenuated lipid accumulation and decreased cell death. Moreover, the use of a PPARα agonist in WT mice prior to ischaemia‐reperfusion injury reduced TIF severity. The authors proposed that the period of activation, the activation status of ATF6 or a cell‐specific context could all explain the opposite roles observed for ATF6 in mice kidney and liver.

## 4 CONCLUSION

**FIGURE 3:**
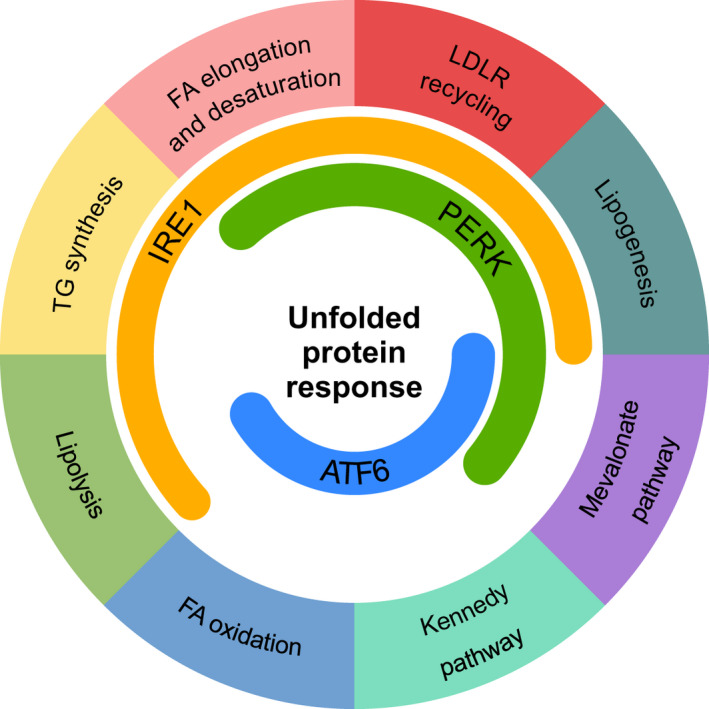
The UPR and lipid metabolism pathways. Overview of how the three arms of the UPR interact with the major pathways involved in lipid metabolism. IRE1 signalling has the most diverse functions in lipid metabolism including lipolysis, triacylglycerol (TG) synthesis, fatty acid (FA) elongation and desaturation, low‐density lipoprotein receptor (LDLR) recycling and lipogenesis. The PERK pathway has been reported to regulate FA elongation and desaturation, LDLR recycling, lipogenesis and mevalonate pathway. ATF6 is mostly linked to lipogenesis, mevalonate pathway and FA oxidation

Besides its well‐characterized role in protein homeostasis, the contributions of the UPR to lipid metabolism are beginning to be appreciated. Multiple studies support the involvement of each of the three branches of the UPR in the modulation of lipid metabolism (Figure 3), and the implications of this in understanding the role of lipid metabolism in restoring ER proteostasis merit further investigation. As the UPR is able to promote both lipogenesis and lipolysis in response to particular cellular contexts and stimuli, its impact on lipid metabolism goes far beyond the classical view of the UPR‐induced activation of ER membrane expansion. While we have tried to summarize here the current knowledge regarding the impact of the UPR on lipid metabolism, several questions remain unanswered. There is an intriguing bidirectional relationship between the UPR and lipid regulation, and we still do not fully understand the operation of the complex feedback mechanisms that support lipid and protein homeostasis in cells. To date, the impact of the UPR on lipid metabolism has largely been examined using chemical inducers of ER stress, which act by inducing a disturbance in protein homeostasis. Given the recent evidence that the ER stress transducers use different mechanisms to sense lipid bilayer stress and proteotoxic stress, and furthermore, that these stresses induce different transcriptional and non‐transcriptional programmes, the interpretation of data using such chemical approaches may need to be re‐evaluated.^100^, ^101^ ER‐mitochondria contact sites (at mitochondria‐associated membranes or MAMs) may be important in the regulation of lipid metabolism by the UPR. MAMs are known to act as sites for lipid trafficking between these two organelles,^102^ and to attenuate IRE1 activity.^103^ Furthermore, there are several proteins that interact with IRE1, comprising the UPRosome, fine‐tuning its function in different ways.^104^ The effect of these proteins on UPR regulation of lipid metabolism is not known.

There are large gaps in our understanding of the relationship between the UPR, lipid metabolism and disease. Many examples of human disease models associated with impaired lipid homeostasis have also been reported to exhibit UPR activation, including atherosclerosis,^105^ type 2 diabetes,^106^ liver disease,^107^ obesity^108^ and cancer.^109^ Taking cancer as an example, ACC^110^ and FASN^111^ have been independently linked to cancer disease progression and regulation of the UPR, while alterations in the mevalonate pathway and cholesterol metabolism have been linked to oncogenesis,^112^ a pathway known to be associated with UPR regulation.^88^, ^105^ These are examples linking lipid metabolism and the UPR or linking cancer and the UPR. However, there are relatively few data linking all three of these. One notable example reports that Myc‐overexpressing cancer cells require SCD1 for sustained growth, which is regulated by IRE1/XBP1 signalling, thus linking UPR, lipid metabolism and cancer.^109^ We anticipate that for cancer, and indeed other diseases, investigation into the interplay between the UPR, lipid metabolism and disease will be a fruitful area of research. Moreover, since UPR activation can lead to cell type‐specific responses, organ‐specific studies (eg in liver, pancreas, kidneys and white adipose tissue) will be informative in developing therapeutic approaches based on modulation of UPR sensors. To that end, new in vivo models such as the recently developed KINGS Ins2^+/G32S^ mouse model of human diabetes will be invaluable.^113^

Taken together, this review highlights a crucial role for UPR in the coordination of lipid metabolism and metabolic reprogramming and suggests this is an important area for further research in relevant diseases.

## CONFLICT OF INTEREST

AS and AG are co‐founders and directors of Cell Stress Discoveries Ltd.

## AUTHOR CONTRIBUTIONS

**Matthieu Moncan:** Visualization (equal); writing‐original draft (lead); writing‐review and editing (equal). **Katarzyna Mnich:** Visualization (equal); writing‐review and editing (equal). **Arnaud Blomme:** Writing‐original draft (equal); writing‐review and editing (equal). **Aitor Almanza:** Writing‐review and editing (equal). **Afshin Samali:** Conceptualization (equal); funding acquisition (equal); writing‐review and editing (equal). **Adrienne M. Gorman:** Conceptualization (equal); funding acquisition (equal); writing‐review and editing (equal).

## Data Availability

Data sharing is not applicable to this article as no new data were created or analysed in this study.
